# An unusual case of ureteric stricture post robotic partial nephrectomy of a renal mass managed by Memokath insertion

**DOI:** 10.1016/j.radcr.2024.05.072

**Published:** 2024-06-22

**Authors:** Ahmed Haroon, Nagy Younes, Khalid El-Sayid, Malkan Khatib, Ibrahim A. Khalil, Jamil Ahmad, Omar M. Abomarzouk, Khalid Al-Rumaihi, Abdulla Al-Ansari

**Affiliations:** a Department of Urology, Hamad Medical Corporation, Doha, Qatar; b Department of Surgery, Surgical Research Section, Hamad Medical Hospital, Hamad Medical Corporation, Doha, Qatar; c College of Medicine, Qatar University, Doha, Qatar; d The University of Medicine, Veterinary and Life Science, University of Glasgow, Scotland, UK

**Keywords:** Memokath, Robotic assisted partial nephrectomy, Ureteric stricture, Small renal mass, Iatrogenic stricture, Case report

## Abstract

Robotic assisted partial nephrectomy is the gold standard treatment for small renal masses. Ureteric stricture is a rare but serious complication that significantly increase the morbidity and worsens the quality of life for cancer patients. Definitive treatment such as surgical reconstruction or ureteroureterostomy is not always feasible as in patients with significant morbidity or high-risk patients. Other options include ureteric double J stent or nephrostomy tube placement with regular exchange. We present a case of iatrogenic upper ureteric stricture post robotic assisted partial nephrectomy for right renal mass that was discovered on postoperative follow up imaging treated with metallic ureteral stent (Memokath) as reconstructive surgery was difficult due to proximity to the tumor bed. We found that if reconstructive surgery is not feasible, metallic ureteral stents has good durability, better quality of life than ureteric double stents for the management of ureteric stricture.

## Introduction

Renal cancers represent 2% to 3% of all cancers, and their incidence is rising. The increased use of ultrasonography and cross-sectional imaging has resulted in the clinical dilemma of incidentally detected small renal masses (SRMs) [1]. The management of SRMs has changed dramatically over the last 2 decades as our understanding of tumor biology and competing risks of mortality in this population has improved. The American Urology Association (AUA) and the European Association of Urology (EAU) guidelines state that PN is the standard of care for renal cell carcinoma (RCC) T1a lesions and should be carried out when technically feasible for T1b renal tumors [2].

Although Robotic-assisted partial nephrectomy (RAPN) appears to be the preferred treatment for selected renal tumors, there are notable complications in up to 35% of cases [3]. The vast majority are classified as low-grade complications, with 50% being medically related [4]. High-grade complications, where an intervention is required, have an incidence of 6%-8% after RAPN [5]. Although rare ureter-associated complications such as ureteric stricture can cause devastating effects of renal function if was not recognized on follow up and treated accordingly. The management of such complications usually involves surgical reconstruction, which carries its complications, but the development of stents, such as metallic stents (Memokath), offers other treatment options with minimal morbidity. We present a case of ureteric stricture post-RAPN that was managed successfully with Memokath insertion.

## Case presentation

**Fig. 1:**
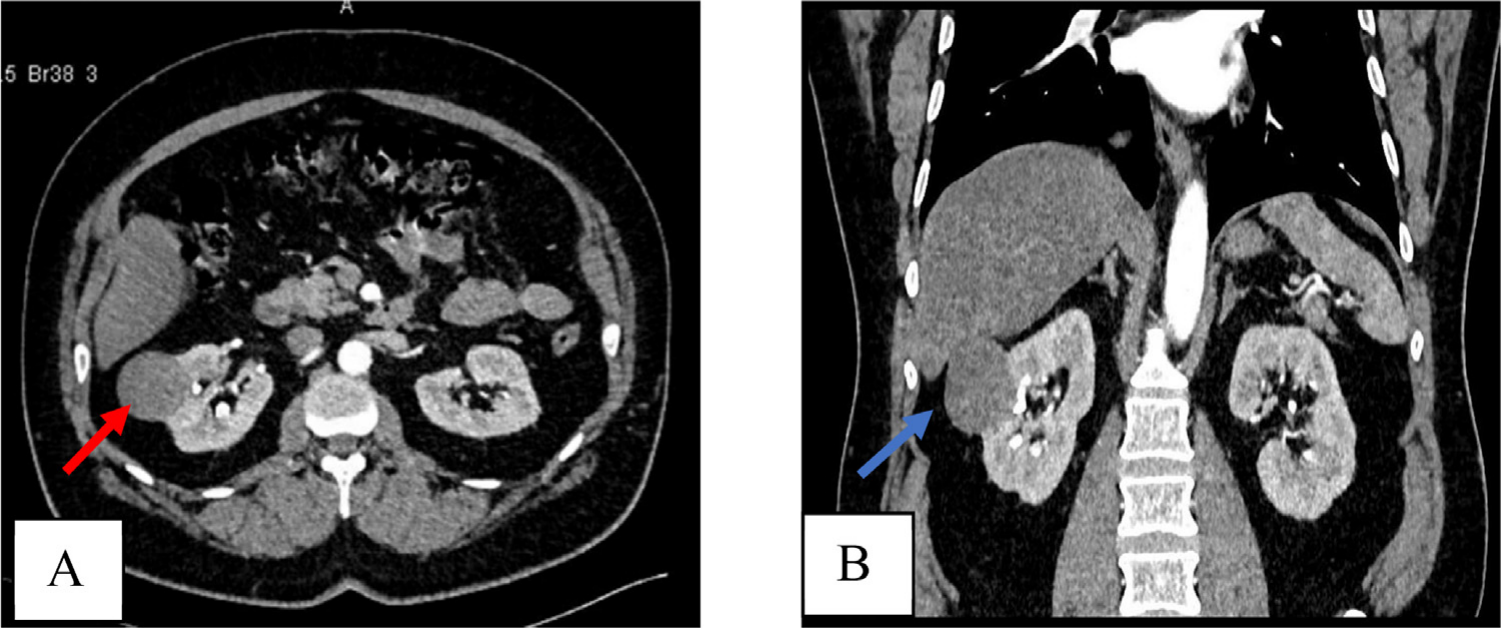
Contrasted CT scan showing a 5 cm mass in the right kidney on (A) cross sectional plane (red arrow) and (B) Coronal section (blue arrow).

A 54-year-old gentleman, known to be diabetic with no past surgical history, underwent robotic-assisted right partial nephrectomy for a 5 cm renal mass diagnosed on computed tomography (CT) scan (Fig. 1). The surgery was uneventful with a warm ischemia time of 25 minutes. Histopathology showed Papillary RCC type 1, with negative margins (pT1a).

**Fig. 2:**
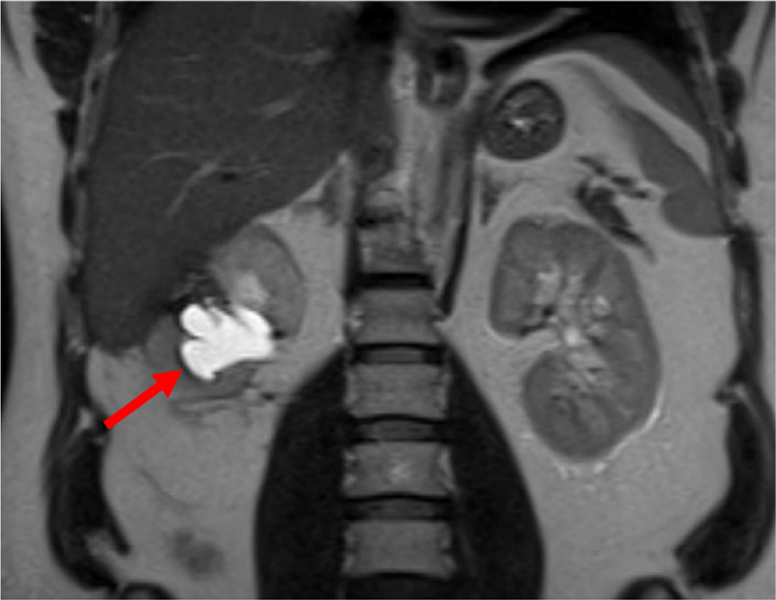
Coronal view MR-Urogram showing right hydronephrosis (red arrow).

**Fig. 3:**
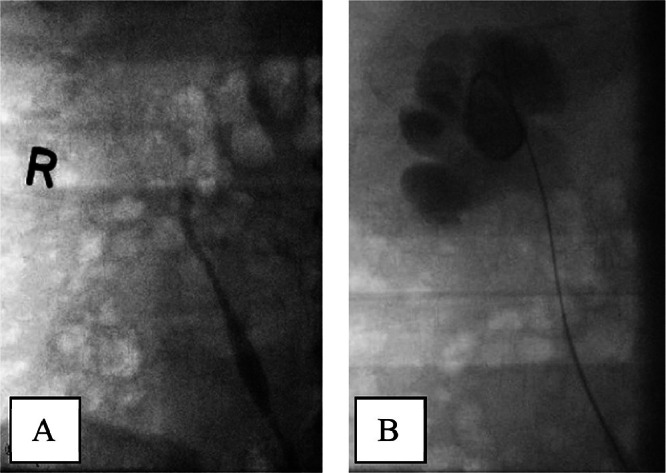
Right retrograde pyelography showing, A: cutoff of contrast at the level of upper ureter, B: Stricture passed by guidewire but not by DJ stent.

**Fig. 4:**
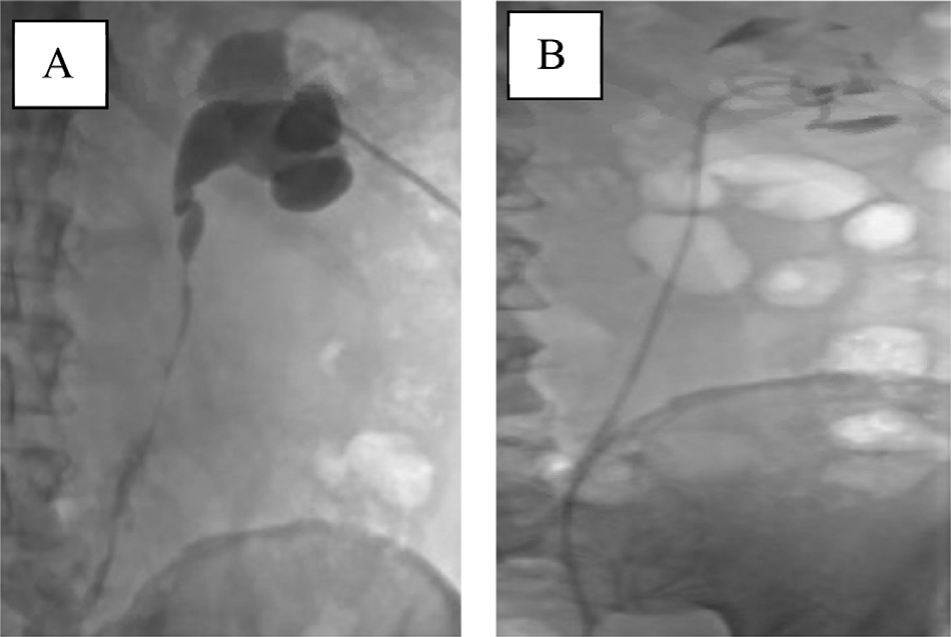
(A) Right Nephrostogram showing right upper ureteric stricture with pre stenotic dilation. Followed by (B) Right Antegrade stenting.

Upon follow-up 6 months postoperation, the patient was asymptomatic. However, his MR urogram showed dilatation of the right pelvicalyceal system and proximal right ureter with poor contrast excretion and enhancement of the right kidney (Fig. 2), along with linear enhancing soft tissue at the proximal ureter likely related to scarring from surgery. Nevertheless, there was no evidence of tumor recurrence. The Tc-99-MAG 3 with lasix, renal scintigraphy showed obstruction of the right kidney and a split function of 20%. The patient was then planned for endoscopic management of the ureteric stricture. Intraoperative retrograde pyelogram showed near-complete obstruction at the level of the upper ureter (Fig. 3), and ureteroscopy using a 4.5 Fr ureteroscope showed a narrow ureter with multiple kinks, confirming the diagnosis of ureteric stricture. A trial of retrograde ureteric catheter failed as it could not pass the stricture. As a result, a right percutaneous nephrostomy tube (PCN) was inserted, followed by antegrade balloon dilation of the ureteric stricture and insertion of a 6/26Fr Double J ureteric catheter (Fig. 4). The patient complained of catheter-related discomfort, such as irritative urinary symptoms, hematuria, and flank pain. After a discussion of the available treatment options, the patient underwent right retrograde ureteric dilation and MemoKath stent insertion four months after the DJ stent insertion. Upon follow-up, the patient was asymptomatic, and the Tc-99-MAG 3 with lasix, renal scintigraphy renogram showed an improvement in right kidney split function to 26%.

### Procedure description

#### Ureteral dilatation and Memokath insertion

Under spinal anesthesia and in lithotomy position, Right double J was removed and then we cannulated the right ureter using sensor guidewire and a 5F open ended catheter. RGP showed complete blockage of the upper ureter and contrast is not reaching the kidney then 4.5 fr semirigid ureteroscope inserted confirmed complete obstruction at the upper ureter.

**Fig. 5:**
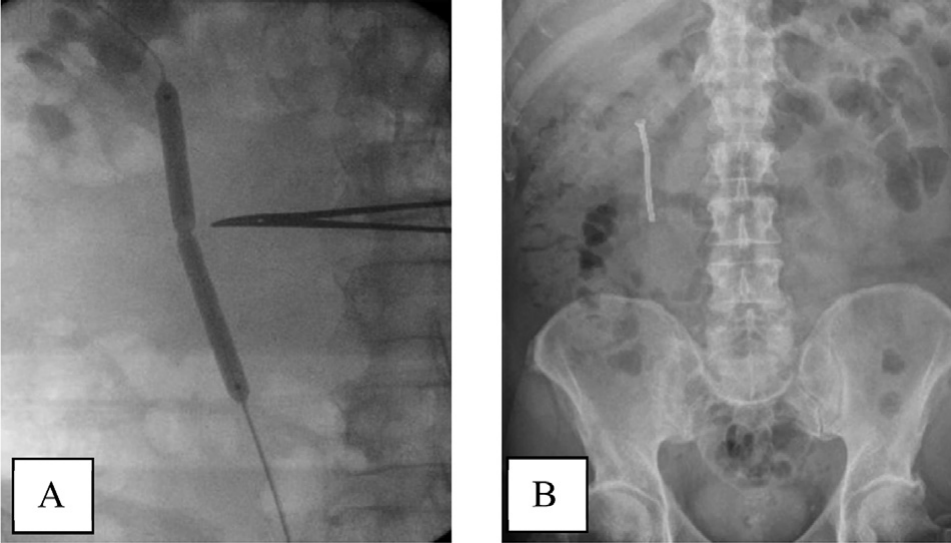
(A) Balloon dilatation of right upper ureteric stricture, followed by (B) Memokath insertion.

We used second sensor guide wire to bypass the upper ureteric stricture, followed by Balloon dilatation (Fig. 5A) and fixation of a 60 mm long Memokath. Position was confirmed by fluoroscopy (Fig. 5B).

## Discussion

As is known within the urologic community, Iatrogenic ureteral stricture post partial nephrectomy is a rare complication of robotic-assisted nephron-sparing surgery (NSS), with an incidence similar to that of laparoscopic NSS. The need for an innovative solution to long-term stenting and reduction of stent-related symptoms resulted in a variety of proposed new stents, out of which metallic ureteric stents, like Memokath, have recently gained more popularity since they emerged as a solution to iatrogenic ureteric stricture, demonstrating good outcomes in a constant trend [6].

The significance of our case started with the incidental finding of the ureteric stricture and deteriorating right kidney function on MRI and renogram on a 6-month follow-up. It required a prompt intervention in order to preserve his kidney function from further deterioration. In these cases, the options are many, with the gold standard being reconstructive surgery such as ureteroureterostomy or ileal transposition for long strictures. In our case, due to the delayed presentation, the risk of adhesions distorting the anatomy and proximity of the stricture to the renal bed, which increase the difficulty and complication of the constructive surgery for a kidney with decreased function, resulted in an alternative approach, such as stenting cares a good prognostic factor with minimal morbidities. Taking into consideration different types of ureteric stenting such as double J stent with regular changes, nephrostomy tube, which also necessitate regular changes, and metallic ureteric stents, we opted to repeat our old experience and use our skills to utilize the option of long-term memokath stenting, which until the date has proven successful [7].

In our institution, this marks another successful treatment for iatrogenic stricture using the memokath, albeit the preceding surgery is different [7]. This intervention has since become more accepted within our institution. It is becoming a main treatment option due to its safety and economic viability for both the patients and the institution in the long term, which further replicates the conclusion in a recent systematic review [8]. This equates to reduced need for in-patient admissions, surgical interventions, total care for potential side effects, and the need for re-stenting [9].

Moreover, memokath is backed up by the 2022 NICE guideline recommendations for patients who potentially require long-term stenting [10]. Yet, while those recommendations are relatively conservative. An emerging literature report on using Memokath in patients with various ureteric strictures of different etiologies [10], which leaves a big room for further trials. A notable study published in 2001 demonstrated the use of the memokath stent with 7 significantly different disease backgrounds in 11 patients. The study concluded a considerable success in the treatment of ureteric strictures, with the only caution advised was the risk of stent encrustation [11].

Due to the construction of the memokath, it has the benefit of reduced irritation and tissue ingrowth compared to the usual double J stents [12]. It is a thermo-expandable stent made from nickel–titanium alloy that has a propensity to close in a tight spiral structure that prevents urothelial ingrowth, preserves peristalsis, and reduces the risk of secondary ischemic damage to the ureteric wall [13].

Although stent migration has been reported [14]. We estimate that with experience and improved suiting of the memokath properties (size, shape, and length), insertion style, and patient selection, the incidence of such side effects will significantly reduce.

The major advantage of the memokath over the standard stenting treatment is that, unlike the double J stent, it can be placed in situ for many years compared to 4-6 months [14]. Some case reports report up to 8 years of in-situ placement with improved quality of life and few patient complaints [15]. This makes the memokath a convenient and surgically sound option for patients who are at risk of redeveloping stricture or ureteral fibrosis. Moreover, in patients with a high risk of developing stones or complex strictures, one study found that memokath stenting may help avoid up to 64% of multiple interventions [16]. Furthermore, Through approaching patients through a questionnaire-based study, patients with memokath stents reported a significantly improved quality of life and a reduction in stent-related symptoms [17].

In our case, we opted to use a metallic stent as the renal function was low to avoid major reconstructive surgery and maximize the benefits with minimal morbidity.

## Conclusion

Memokath ureteric stent is an excellent, cost-effective alternative to other stenting options like double J stents in treating iatrogenic ureteric strictures whenever reconstructive surgeries are not feasible. They provide better outcomes with less morbidity, side effects, and reduced costs.

## Patient consent

Informed consent was obtained from the patient according to our hospital policy.
